# Long-distance spread of Tembusu virus, and its dispersal in local mosquitoes and domestic poultry in Chongming Island, China

**DOI:** 10.1186/s40249-023-01098-9

**Published:** 2023-05-22

**Authors:** Yuan Fang, Tian Hang, Li-Min Yang, Jing-Bo Xue, Ryosuke Fujita, Xue-Song Feng, Tian-Ge Jiang, Yi Zhang, Shi-Zhu Li, Xiao-Nong Zhou

**Affiliations:** 1 School of Global Health, Chinese Center for Tropical Diseases Research, Shanghai Jiao Tong University School of Medicine, Shanghai, China; 2 National Institute of Parasitic Diseases, Chinese Center for Disease Control and Prevention (Chinese Center for Tropical Diseases Research); NHC Key Laboratory of Parasite and Vector Biology; WHO Collaborating Centre for Tropical Diseases; National Center for International Research on Tropical Diseases,, Shanghai, China; 3 Laboratory of Sanitary Entomology, Faculty of Agriculture, Kyushu University, Fukuoka, Japan; 4 Shanghai Chongming Dongtan National Nature Reserve, Shanghai, China

**Keywords:** Chaoyang virus, Emerging zoonotic vector-borne disease, Insect-specific flavivirus, Mosquito-borne virus, Quang Binh virus

## Abstract

**Background:**

Chongming Island in China serves as a breeding and shelter point on the East Asian–Australasian Flyway. The resting frequency of migratory birds, abundance of mosquito populations, and the popular domestic poultry industry pose a potential risk of mosquito-borne zoonotic diseases. The aim of this study is to explore the role of migratory birds in the spread of mosquito-borne pathogens and their prevalent status on the island.

**Methods:**

We conducted a mosquito-borne pathogen surveillance in 2021, in Chongming, Shanghai, China. Approximately 67,800 adult mosquitoes belonging to ten species were collected to investigate the presence of flaviviruses, alphaviruses, and orthobunyaviruses by RT-PCR. Genetic and phylogenetic analyses were conducted to explore the virus genotype and potential nature source. Serological survey was performed by ELISA to characterize Tembusu virus (TMUV) infection among domestic poultry.

**Results:**

Two strains of TMUV and Chaoyang virus (CHAOV) and 47 strains of Quang Binh virus (QBV) were detected in 412 mosquito pools, with the infection rate of 0.16, 0.16, and 3.92 per 1000 *Culex tritaeniorhynchus*, respectively. Furthermore, TMUVs viral RNA was found in serum samples of domestic chickens and faecal samples of migratory birds. Antibodies against TMUV were detected in domestic avian serum samples, generally ranging from 44.07% in pigeons to 55.71% in ducks. Phylogenetic analyses indicated that the TMUV detected in Chongming belonged to Cluster 3, Southeast Asia origin, and most closely related to the CTLN strain, which caused a TMUV outbreak in chickens in Guangdong Province in 2020, but distant from strains obtained previously in Shanghai, which were involved in the 2010 TMUV outbreak in China.

**Conclusions:**

We speculate that the TMUV was imported to Chongming Island through long-distance spreading by migratory birds from Southeast Asia, followed by spill over and transmission in mosquitoes and domestic avian species, threatening the local domestic poultry. In addition, the expansion and prevalence of insect-specific flaviviruses and its simultaneous circulation with mosquito-borne virus are worthy of close attention and further study.

**Supplementary Information:**

The online version contains supplementary material available at 10.1186/s40249-023-01098-9.

## Background

The epidemic of emerging zoonotic vector-borne diseases poses a serious risk to public health. Zika virus, was previously transmitted by mosquitoes in a sylvatic cycle between non-human primates and it “spilled over” into human transmission cycle through infected mosquito bites to rural or even urban populations resulting in the 2015–2016 pandemic [1]. Tembusu virus (TMUV), another flavivirus was first isolated from *Culex tritaeniorhynchus* mosquitoes in Malaysia in 1955 [2] and the Sitiawan strain of TMUV was isolated from domestic chickens in Malaysia [3]. Thereafter, TMUV was detected in both, mosquitoes and ducks in Thailand in 2002 [4]. Due to its silent transmission, TMUV was neglected in Southeast Asia until its emergence and outbreak in ducks, spreading quickly across south-eastern coastal provinces and neighbouring regions of China, causing a series of epidemics in the duck farming industry since April 2010 [5, 6], sporadically re-emerging, especially in 2012 and 2015. which resulted in tremendous economic losses in the poultry industry in China [7–10]. TMUV outbreaks have also occurred in several duck farms in Malaysia in 2012 [11], and in Thailand in 2013 [12]. Epidemiological studies have shown that TMUV outbreaks occur in seasons when mosquitoes are inactive, suggesting transmission in absence of the vectors, and subsequent in vivo studies indicated that TMUV can be transmitted efficiently among ducks by both direct contact and aerosol transmission [13, 14].

TMUV causes decline in egg production, ataxia, reluctance to walk, and paralysis in ducks, with a mortality rate of up to 90% in infected shelduck layer farms [15] and morbidity rate due to secondary bacterial infections varying from 5 to 15% [6]. Circulation of TMUV has been restricted to Malaysia, Thailand, and China [3, 5, 16]. In China, it has specifically spread to 19 provinces/autonomous regions/municipalities (P/A/M) including Anhui, Beijing, Chongqing, Fujian, Guangdong, Guangxi, Hebei, Henan, Hubei, Hunan, Inner Mongolia, Jiangsu, Jiangxi, Shandong, Shanghai, Sichuan, Taiwan, and Yunnan [5, 17].

*Culex* mosquitoes involved in the transmission of TMUV, include *Cx. tritaeniorhynchus*, *Cx. vishnui*, *Cx. gelidus* [4, 5, 16], *Cx. pipiens* [8], *Cx. pipiens quinquefasciatus* [18], and *Cx. annulus* [17]. TMUV is also detected in avian species including ducks [7, 15], chickens [3, 10], geese [19], pigeons [20], and house sparrows [21]. A high seroprevalence (71.9%) of TMUV antibodies and viral RNA has been detected in duck farm workers with known TMUV infection outbreaks in Shandong Province, China [22].

Chongming District, composed of Chongming Island, Changxing and Hengsha islands, is the ecological barrier of Shanghai Municipality. The Dongtan Nature Reserve of Chongming Island located at the mouth of the Yangtze Estuary is an important stop point and wintering inhabit for millions of migratory birds each year, on the East Asian–Australasian Flyway, which passes through countries like Australia, Malaysia, Thailand, China, and the Russian Far East. Distributed across the East to the West of Chongming Island is its ecological and sustainable poultry industry which includes duck, chicken, and pigeon farming. Migratory birds are known to contribute to the dispersal of zoonotic pathogens into new areas that are distant from their origins [23]. Undoubtedly, the emergence and re-emergence of pathogens carried by migratory birds pose a great challenge to the local domestic poultry industry.

There is no official documentation of TMUV-like symptoms by the Shanghai Chongming District Animal Disease Prevention and Control Center in Chongming Island and, to the best of our knowledge, studies on TMUV in this island are scarce. The mosquito population is diverse and abundant in Chongming, facilitating the spill over of avian mosquito-borne viruses into domestic birds [24]. In our previous survey on mosquito-borne diseases in Shanghai with field caught mosquitoes, two insect-specific flaviviruses (ISFVs), Culex flavivirus and Quang Binh virus (QBV), were first recorded in Chongming [25]; however, no mosquito-borne zoonotic pathogens have been detected, probably due to uneven distribution and a limited number of sentinel sites. Therefore, it remains unclear whether TMUV or other mosquito-borne zoonotic viruses circulate in Chongming, an important stopover and wintering site for migrating birds with vector abundance and popular poultry industry. It is important to study the role of migratory birds in the spread of emerging pathogens and their prevalent status on the island. In this study, mosquito and poultry samples were analysed to investigate the species, genotype, spreading model, mode of transmission of mosquito-borne viruses, and their prevalence in domestic poultry in Chongming Island.

## Methods

### Mosquito collection

**Fig. 1:**
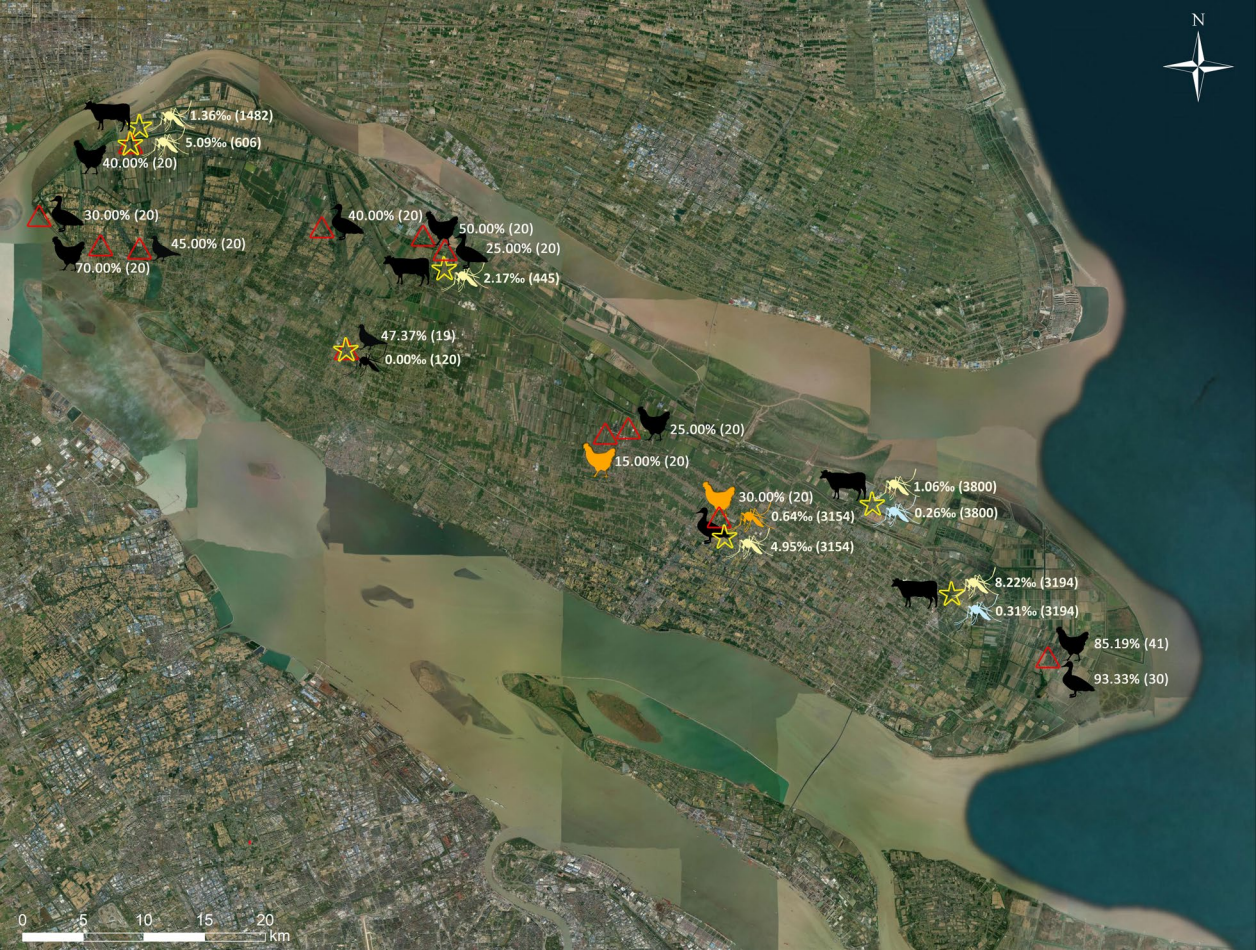
The distribution of mosquito-borne viruses and seroprevalence of Tembusu virus (TMUV) antibodies across Chongming Island, Shanghai, China. Stars represent collection sites of mosquito specimens. Mosquito symbols filled with orange, light yellow, and light blue, represent positive for Tembusu virus (TMUV), Chaoyang virus (CHAOV), and Quang Binh virus (QBV) in Culex tritaeniorhynchus, respectively. The infection rates of mosquito-borne viruses with sample size in each collection sites are shown at behind. Stars represent the collection sites of avian serum samples. The seropositive rates of TMUV-antibody with sample size in each domestic poultry farm are shown behind avian symbols. Chicken symbol filled with orange represent RNA positive for TMUV.

Mosquitoes were collected during the mosquito activity season from May to October, 2021, using traps with ultraviolet light (Wuhan Lucky Star Environmental Protection Technology Co., Ltd., Wuhan, China), for two consecutive nights each month, by hanging them from sunset to sunrise for overnight collections. A total of seven sentinel sites were selected along the island in three directions, east, central, and west, representing areas close to the gateway to the mainland of Shanghai, suburban areas, and intensive farming areas. One domestic poultry and livestock farm from each area was selected for mosquito collection and an extra sentinel site was located near the Dongtan National Nature Reserve. The geographic location of each sentinel site is shown in Fig. 1 and was generated using ArcGIS 10.1 ArcMap software (ESRI, Redlands, CA, USA). All mosquito samples were kept in an ice bath, identified using morphological characteristics according to the national index [26], and pooled by species, gender, collection date, and location. Each pool consisted of 1–50 mosquitoes which were frozen at − 80 °C until further pathogen detection.

### Poultry sample collection

#### Serum collection

Serum samples of chickens (*Gallus gallus domesticus*), ducks (*Anas platyrhyncha* var. *domestica*), and pigeons (*Columba livia*) were provided by the Shanghai Chongming District Center for Animal Disease Prevention and Control based on their routine surveillance strategy for animal diseases. The collection sites were distributed across the island. Serum samples of domestic poultry were collected from the nearby Dongtan National Nature Reserve for TMUV serological investigation, where close to the habitat of migratory birds.

#### Faecal and saliva sample collection of wild birds

Cloacal and throat swab samples of migratory birds (*A. platyrhyncha*) were collected with the help of the Shanghai Chongming Dongtan National Nature Reserve personnel.

### Virus identification

RNA was extracted from 412 pools of 15,675 mosquitoes (both female and male mosquitoes), from samples captured from almost all the sentinel sites, except from duck and cow farms, which are in the eastern part. We randomly selected samples collected from these two sites for virus detection, as the number of mosquitoes per night per trap exceeded 5000 in July and August. Serum samples and samples of faecal and saliva were pre-mixed with Trizol before vortex, then centrifuged at full speed to separate the supernatant for further RNA extraction by MagNA Pure 96 System (Roche, Basel, Switzerland). RNA extraction for mosquito samples were performed as previously described [25]. First-strand cDNA was synthesized by reverse transcriptase using a PrimeScript RT Reagent Kit with gDNA Eraser (Takara Bio, Shiga, Japan). To assess RNA integrity, the presence of mosquito 18 S rRNA was verified by PCR using synthesized cDNAs [27]. Flavivirus detection was performed using the universal flavivirus primer sets cFD2/MAMD and cFD2/FS778 (by hemi-nested PCR), targeting the partial *NS5* gene [28]. Primer sets for amplification of the full-length TMUV *E* gene were used according to Huang, et al. [7]. Whole genomes of TMUV and QBV were generated as per previous reports [5, 10, 29]. Samples were screened for the presence of alphaviruses and orthobunyaviruses using primer sets α6533f/α6999c [30] and BCS82C/BCS332V [31], respectively. The positive products were purified, cloned, and sequenced by Sangon Biotech (Shanghai, China). To avoid cross-contamination, the avian samples were not processed in parallel with the mosquito samples in the processes both for RNA extraction and PCR. PCR products were visualized on 1.2 or 2% agarose gels using Goldview (Beijing Solarbio Science & Technology Co., Ltd., Beijing, China).

### Phylogenetic analyses

The obtained sequences were compared with those deposited in the GenBank database using BLAST [32]. Available sequences for flavivirus *NS5* (> 200 bp), and *E* gene (~ 1500 bp) of TMUV were downloaded from GenBank database. Sequences representative of different geographic origin (countries, provinces), different host, and incriminated in TMUV outbreaks were selected for further phylogenetic analyses. Multiple sequence alignments were generated with ClustalW2 with default settings, and manually adjusted as required [33]. Neighbor-joining (NJ) trees were plotted using Kimura’s two-parameter (K2P) distance model [34] and 1000 bootstrap replicates in MEGA v. 7.0 [35]. Based on the Akaike Information Criterion, a best-fit alignment model was determined using Modeltest v. 3.7 and PAUP* v. 4.0b10 [36]. The maximum likelihood (ML) and Bayesian likelihood trees were plotted using the GTR + I + G model for *NS5* gene, and *E* gene of TMUV. The ML and Bayesian likelihood trees were plotted using the TIM + G and TrN + I + G models for *NS5* genes of Chaoyang virus (CHAOV) and QBV, respectively. The ML trees were generated with MEGA v. 7.0 using 1000 bootstrap replicates. The Bayesian trees were constructed using MrBayes v. 3.2.1 [37] and run for ten million generations, of which 25% were discarded as burn-in. The trees were visualized using Figtree v. 1.4.2 (http://tree.bio.ed.ac.uk/software/figtree/).

### Comparison of virus deduced amino acid sequences

The *E* genes of TMUVs sequenced in this study were translated into amino acid sequences and aligned with the FX2010 strain involved in 2010 TMUV outbreaks [15] and the live attenuated vaccine strain FX2010-180P derived from FX2010 [38], using MEGA v. 7.0.

### Infection rate

As the sizes of the pools of collected mosquitoes varied considerably, infection rates were calculated by bias-corrected maximum likelihood estimation (MLE) and minimum infection rate (MIR) using the Excel add-in PooledInfRate v.4 statistical software package [39] and were expressed as the number of infected mosquitoes per 1000 individuals.

### Serological survey

The serum samples of domestic poultry were screened for the presence of TMUV antibodies using blocking ELISA [40]. Briefly, 10 µl of serum from each sample was used for the assay, and the test procedure was completed according to the manufacturer’s protocol. The colour change was measured spectrophotometrically 10 min after stopping the reaction at 450 nm using an absorbance microplate reader (Multiskan FC, Thermo Scientific, China).

## Results

### Detection of mosquito-borne pathogens

#### Mosquito samples

In total, 67,800 adult mosquitoes belonging to ten species from four genera of the family Culicidae (*Culex*, *Aedes*, *Anopheles*, and *Armigeres*) were collected from seven survey sites (Fig. 1) between June and October 2021 in Shanghai. Among them, *Cx. tritaeniorhynchus, An. sinensis*, and *Ae. vexans* accounted for 96.41% (65,363/67,800), 2.70% (1830/67,800), and 0.64% (435/67,800), respectively. Seven other species, *Cx. pipiens pallens*, *Cx. bitaeniorhynchus*, *Cx. modestus*, *Ae. dorsalis*, *Ae. togoi*, *Ae. albopictus*, and *Ar. subalbatus* were collected but in extremely limited numbers. Fifty-one mosquito pools were positive for the partial flavivirus *NS5* gene, including two TMUVs, two CHAOVs, and 47 QBVs. All positive results were observed in female mosquito pools. Neither alphaviruses nor orthobunyaviruses were detected in this study. *E* gene of two TMUV strains, whole genome of one TMUV and three QBV strains, were successfully amplified. The virus name, host species, collection date, inhabitants, and corresponding GenBank accession numbers of the virus strains obtained in this study are shown in Table 1.

**Table 1:** Summary of the flaviviruses detected from mosquito and avian samples, obtained in Chongming Island, Shanghai in 2021

Strain	Virus	Vector/ Host	Collection date	Inhabitant	GenBank ID		
					*NS5*	*E*	Whole genome
Mosquito							
21-9-DY-CXT-5	TMUV	*Culex tritaeniorhynchus*	Sep-21	Poultry farm^a^	OP087424	OP104342	
21-9-DY-CXT-9	TMUV	*Cx. tritaeniorhynchus*	Sep-21	Poultry farm^a^	OP087425		
21-6-NF1-CXT-1	CHAOV	*Cx. tritaeniorhynchus*	Jun-21	Buffalo farm^a^	OP087426		
21-7-SY-CXT-12	CHAOV	*Cx. tritaeniorhynchus*	Jul-21	Cow farm^a^	OP087427		
21-7-NF1-CXT-1	QBV	*Cx. tritaeniorhynchus*	Jul-21	Buffalo farm^a^	OP087428		
21-7-NF1-CXT-2-1	QBV	*Cx. tritaeniorhynchus*	Jul-21	Buffalo farm^a^	OP087429		
21-7-NF1-CXT-2-2	QBV	*Cx. tritaeniorhynchus*	Jul-21	Buffalo farm^a^	OP087430		
21-7-NF1-CXT-3	QBV	*Cx. tritaeniorhynchus*	Jul-21	Buffalo farm^a^	OP087431		
21-7-NF1-CXT-5	QBV	*Cx. tritaeniorhynchus*	Jul-21	Buffalo farm^a^	OP087432		
21-7-NF1-CXT-8	QBV	*Cx. tritaeniorhynchus*	Jul-21	Buffalo farm^a^	OP087433		
21-7-NF1-CXT-10-1	QBV	*Cx. tritaeniorhynchus*	Jul-21	Buffalo farm^a^	OP087434		
21-7-NF1-CXT-10-2	QBV	*Cx. tritaeniorhynchus*	Jul-21	Buffalo farm^a^	OP087435		
21-7-NF1-CXT-11	QBV	*Cx. tritaeniorhynchus*	Jul-21	Buffalo farm^a^	OP087436		OP087418
21-7-NF1-CXT-13	QBV	*Cx. tritaeniorhynchus*	Jul-21	Buffalo farm^a^	OP087437		
21-7-NF1-CXT-14-1	QBV	*Cx. tritaeniorhynchus*	Jul-21	Buffalo farm^a^	OP087438		
21-7-NF1-CXT-14-2	QBV	*Cx. tritaeniorhynchus*	Jul-21	Buffalo farm^a^	OP087439		
21-7-NF1-CXT-15	QBV	*Cx. tritaeniorhynchus*	Jul-21	Buffalo farm^a^	OP087440		
21-7-NF1-CXT-16	QBV	*Cx. tritaeniorhynchus*	Jul-21	Buffalo farm^a^	OP087441		
21-7-NF1-CXT-18	QBV	*Cx. tritaeniorhynchus*	Jul-21	Buffalo farm^a^	OP087442		OP087419
21-7-NF1-CXT-20	QBV	*Cx. tritaeniorhynchus*	Jul-21	Buffalo farm^a^	OP087443		
21-7-NF1-CXT-21	QBV	*Cx. tritaeniorhynchus*	Jul-21	Buffalo farm^a^	OP087444		
21-7-NF1-CXT-22	QBV	*Cx. tritaeniorhynchus*	Jul-21	Buffalo farm^a^	OP087445		
21-9-NF1-CXT-1	QBV	*Cx. tritaeniorhynchus*	Sep-21	Buffalo farm^a^	OP087446		
21-9-NF1-CXT-3	QBV	*Cx. tritaeniorhynchus*	Sep-21	Buffalo farm^a^	OP087447		
21-9-NF1-CXT-4	QBV	*Cx. tritaeniorhynchus*	Sep-21	Buffalo farm^a^	OP087448		
21-10-NF-CXT-1	QBV	*Cx. tritaeniorhynchus*	Oct-21	Buffalo farm^a^	OP087449		
21-7-SY-CXT-18	QBV	*Cx. tritaeniorhynchus*	Jul-21	Cow farm^a^	OP087450		
21-8-SY-CXT-1	QBV	*Cx. tritaeniorhynchus*	Aug-21	Cow farm^a^	OP087451		
21-8-SY-CXT-3	QBV	*Cx. tritaeniorhynchus*	Aug-21	Cow farm^a^	OP087452		
21-10-SY-CXT	QBV	*Cx. tritaeniorhynchus*	Oct-21	Cow farm^a^	OP087453		
21-9-DY-CXT-4-4	QBV	*Cx. tritaeniorhynchus*	Sep-21	Poultry farm^a^	OP087454		
21-9-DY-CXT-4-5	QBV	*Cx. tritaeniorhynchus*	Sep-21	Poultry farm^a^	OP087455		
21-9-DY-CXT-5-Q	QBV	*Cx. tritaeniorhynchus*	Sep-21	Poultry farm^a^	OP087456		
21-9-DY-CXT-6-3	QBV	*Cx. tritaeniorhynchus*	Sep-21	Poultry farm^a^	OP087457		
21-9-DY-CXT-6-5	QBV	*Cx. tritaeniorhynchus*	Sep-21	Poultry farm^a^	OP087458		
21-9-DY-CXT-8-3	QBV	*Cx. tritaeniorhynchus*	Sep-21	Poultry farm^a^	OP087459		
21-9-DY-CXT-12	QBV	*Cx. tritaeniorhynchus*	Sep-21	Poultry farm^a^	OP087460		
21-9-DY-CXT-19	QBV	*Cx. tritaeniorhynchus*	Sep-21	Poultry farm^a^	OP087461		
21-10-DY-CXT-1-1	QBV	*Cx. tritaeniorhynchus*	Oct-21	Poultry farm^a^	OP087462		
21-10-DY-CXT-1-2	QBV	*Cx. tritaeniorhynchus*	Oct-21	Poultry farm^a^	OP087463		
21-10-DY-CXT-1-3	QBV	*Cx. tritaeniorhynchus*	Oct-21	Poultry farm^a^	OP087464		
21-10-DY-CXT-2	QBV	*Cx. tritaeniorhynchus*	Oct-21	Poultry farm^a^	OP087465		
21-10-DY-CXT-3	QBV	*Cx. tritaeniorhynchus*	Oct-21	Poultry farm^a^	OP087466		
21-10-DY-CXT-5	QBV	*Cx. tritaeniorhynchus*	Oct-21	Poultry farm^a^	OP087467		
21-7-AS-CXT-2	QBV	*Cx. tritaeniorhynchus*	Jul-21	Cow farm^b^	OP087468		
21-7-YJ-CXT-2	QBV	*Cx. tritaeniorhynchus*	Jul-21	Cow farm^c^	OP087469		
21-7-YJ-CXT-5	QBV	*Cx. tritaeniorhynchus*	Jul-21	Cow farm^c^	OP087470		
21-9-YJ-CXT-1	QBV	*Cx. tritaeniorhynchus*	Sep-21	Cow farm^c^	OP087471		
21-9-YY-CXT-2	QBV	*Cx. tritaeniorhynchus*	Sep-21	Chicken farm^c^	OP087472		
21-9-YY-CXT-3	QBV	*Cx. tritaeniorhynchus*	Sep-21	Chicken farm^c^	OP087473		OP087420
21-10-NF-AEX	QBV	*Aedes vexans*	Oct-21	Buffalo farm^a^	OP087474		
Avian							
21-5-GY-8	TMUV	Chicken	May-21	Poultry farm^a^	OP087475	OP104343	OP087421
21-5-GY-20	TMUV	Chicken	May-21	Poultry farm^a^	OP087476		
21-5-DY-11	TMUV	Chicken	May-21	Poultry farm^a^	OP087477		
21-5-DY-7	TMUV	Chicken	May-21	Poultry farm^a^	OP087478		
DT2-G	TMUV	Goose	Dec-21	Poultry farm^a^	OP087479		
bird_G10	TMUV	Migratory Bird	Dec-21	Nature Reserve^a^	OP087480		
DT2-ChickSerum36	QBV	Chicken	Dec-21	Poultry farm^a^	OP087481		
DT1-DuckSerum5	QBV	Duck	Nov-21	Poultry farm^a^	OP087482		
DT1-DuckSerum7	QBV	Duck	Nov-21	Poultry farm^a^	OP087483		
bird_Y6	QBV	Migratory Bird	Dec-21	Nature Reserve^a^	OP087484		

Poultry: mixed flocks of adult chickens and ducks, ^a^: Eastern Chongming Island; ^b^: Central Chongming Island; ^c^: Western Chongming Island

TMUV was present in two pools of *Cx. tritaeniorhynchus* collected from a poultry farm in September 2021 in eastern Chongming, China (Fig. 1). One CHAOV strain was detected in *Cx. tritaeniorhynchus* pool, collected from a buffalo farm near the Nature Reserve in June and a cow farm in eastern region in July. QBVs were found in most farms in all three directions of collection sites but was not detected in a pigeon farm in the central region, probably due to the small sample size. Most of QBV positive were from *Cx. tritaeniorhynchus* pools, with the exception of one *Ae. vexans* pool. Most QBVs were obtained from the buffalo farm, near the Nature Reserve, and the chicken farm in the East of Chongming Island (Fig. 1).

### Serum virus detection

The RNA was extracted from 87, 93, and 48 chicken, duck, and pigeon serum samples, respectively, from different poultry farms to detect the presence of flavivirus. TMUV was positive in each two serum samples obtained from two domestic poultry farms (Fig. 1). In samples obtained from a private poultry farm near the Nature Reserve, TMUV was detected in a goose serum sample, and QBV was found in two duck serum samples and one chicken serum sample. Additionally, TMUV was present in a faecal sample of migratory birds, and a partial *NS5* gene of QBV was successfully amplified in a saliva sample from migratory birds.

### Phylogenetic analyses

#### Tembusu virus

**Fig. 2:**
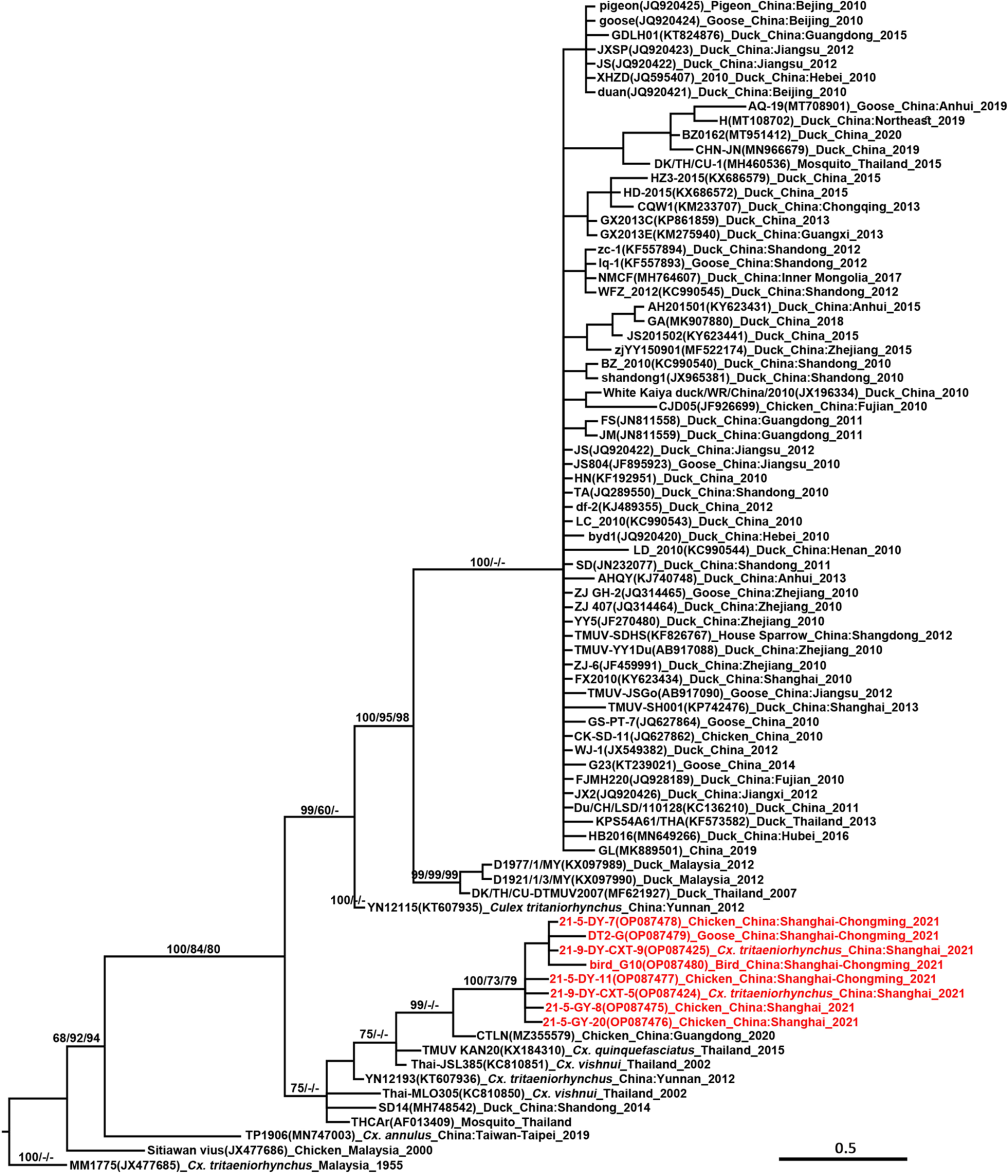
Phylogenetic tree generated by Bayesian analysis of partial non-structural 5 gene sequences of Tembusu virus (TMUV). The virus strain, GenBank accession number, host, collection country, and year are noted. The TMUV sequences obtained in this study are marked in red. Bootstrap values (1000 replicates, not shown for less than 60%) of Bayesian analyses, maximum likelihood, and neighbor-joining are shown above the main lineages. The bar indicates 0.5 substitutions per site

**Fig. 3:**
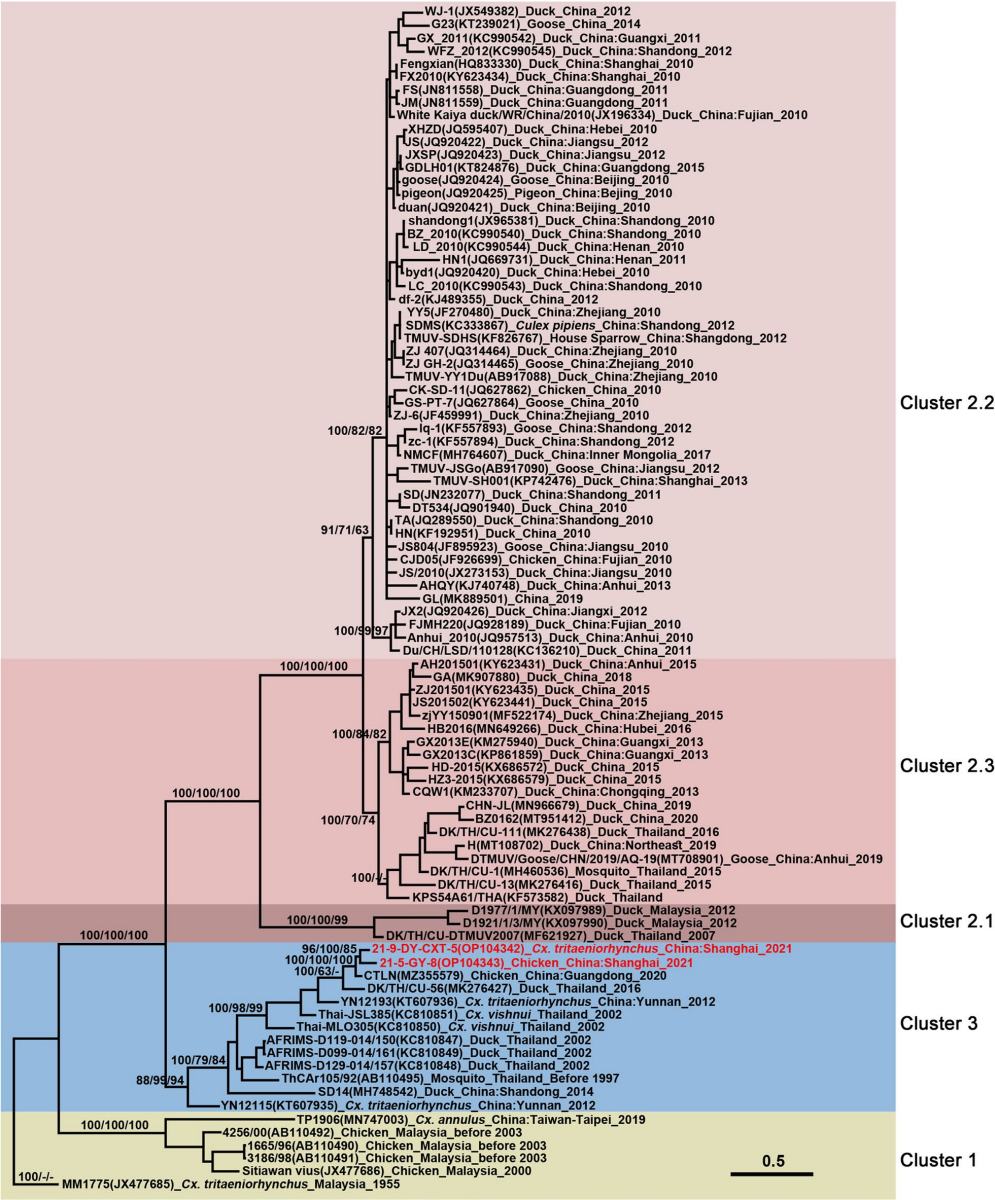
Phylogenetic tree generated by Bayesian analysis of Tembusu virus (TMUV) envelope gene. The virus strain, GenBank accession number, host, collection country, and year are noted. The TMUV sequences obtained in this study are marked in red. Bootstrap values (1000 replicates, not shown for less than 60%) of Bayesian analyses, maximum likelihood, and neighbor-joining are shown above the main lineages. The scale-bar indicates 0.5 substitutions per site. Sequences shaded khaki represent Cluster 1, those shaded rosy brown represent Subcluster 2.1, those shaded misty rose represent Subcluster 2.2, those shaded light coral represent Subcluster 2.3, and those shaded sky blue represent the TMUV Cluster 3

TMUV was detected in both mosquito and avian specimens collected in Chongming and their *NS5* sequences had 97.51–100.00%, and 97.01–100.00% sequence similarity in nucleotide and amino acid sequences, respectively. They were grouped together in the *NS5* tree, rooted by the prototype MM1775 strain (Fig. 2), and were most closely related to the samples obtained from *Culex* spp., originating in Southeast Asia, mainly from Thailand. The *E* gene of each *Cx. tritaeniorhynchus* pool and chicken serum samples shared 99.23% and 99.15% nucleotide and amino acid identities, respectively. They shared 88.90–89.10% similarity at the nucleotide level with FX2010 and TMUV-SH001 strains. Whereas, the nucleotide identity of *E* gene between the CTLN and 21-5-GY-8 strains was 99.37% and their similarity was 99.79% at the amino acid level. The phylogenetic tree based on the *E* gene of TMUV (Fig. 3) showed that TMUV consists of three distinct clusters: Clusters 1, 2, and 3. The newly obtained Chongming TMUV sequences belong to Cluster 3 which mainly contains sequences obtained from mosquito and duck samples collected in Thailand and Yunnan Province of China, in addition to the SD14 strain isolated from mallards in Shandong in 2014 and the CTLN strain isolated from layer hens in Guangdong in 2020. Consistently with the review of *NS5* tree, the *E* gene tree of TMUV showed that the Chongming TMUV strains were far from the previous Shanghai strains but were closely related to strains, mainly from Southeast Asia.

#### Chaoyang virus

**Fig. 4:**
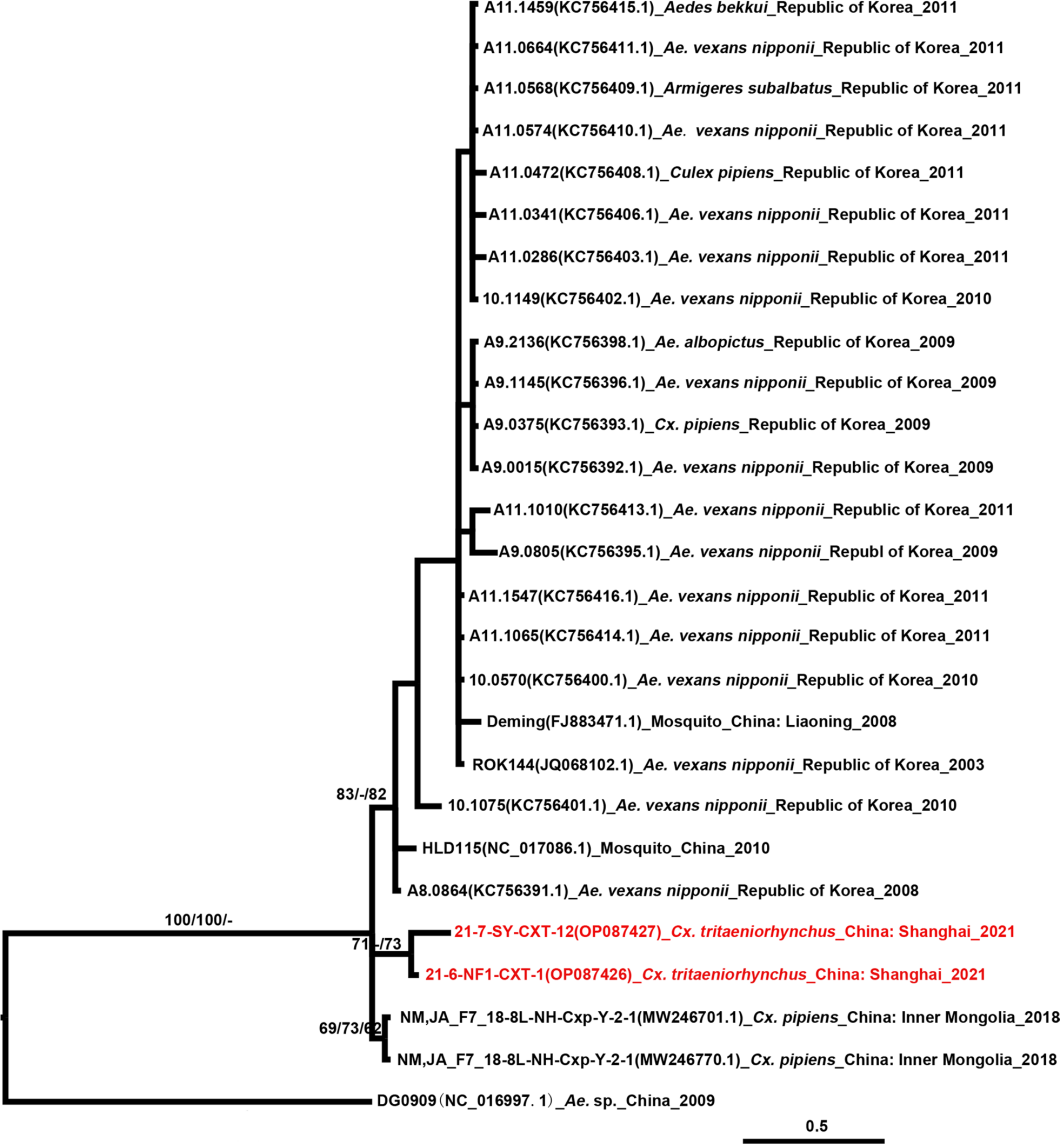
Phylogenetic tree generated by Bayesian analysis of partial non-structural 5 gene sequences of Chaoyang virus (CHAOV). The virus strain, GenBank accession number, host, collection country, and year are noted. The tree was rooted by Donggang virus (DGV, DG0909 strain). The CHAOV sequences obtained in this study are marked in red. Bootstrap values (1−000 replicates, not shown for less than 60%) of Bayesian analyses, maximum likelihood, and neighbor-joining are shown above the main lineages. The bar indicates 0.5 substitutions per site

The two Chongming CHAOV strains shared 100% nucleotide sequence identity with the partial *NS5* gene and 97.13% amino acid sequence identity with strains from *Cx. pipiens* collected from Inner Mongolia in 2018. In view of the topology of the CHAOV *NS5* tree (Fig. 4), the two Chongming strains were monophyletic and distinct from strains obtained in Northeast Asia (Liaoning Province of China, and Republic of Korea) and Inner Mongolia.

#### Quang Binh virus

**Fig. 5:**
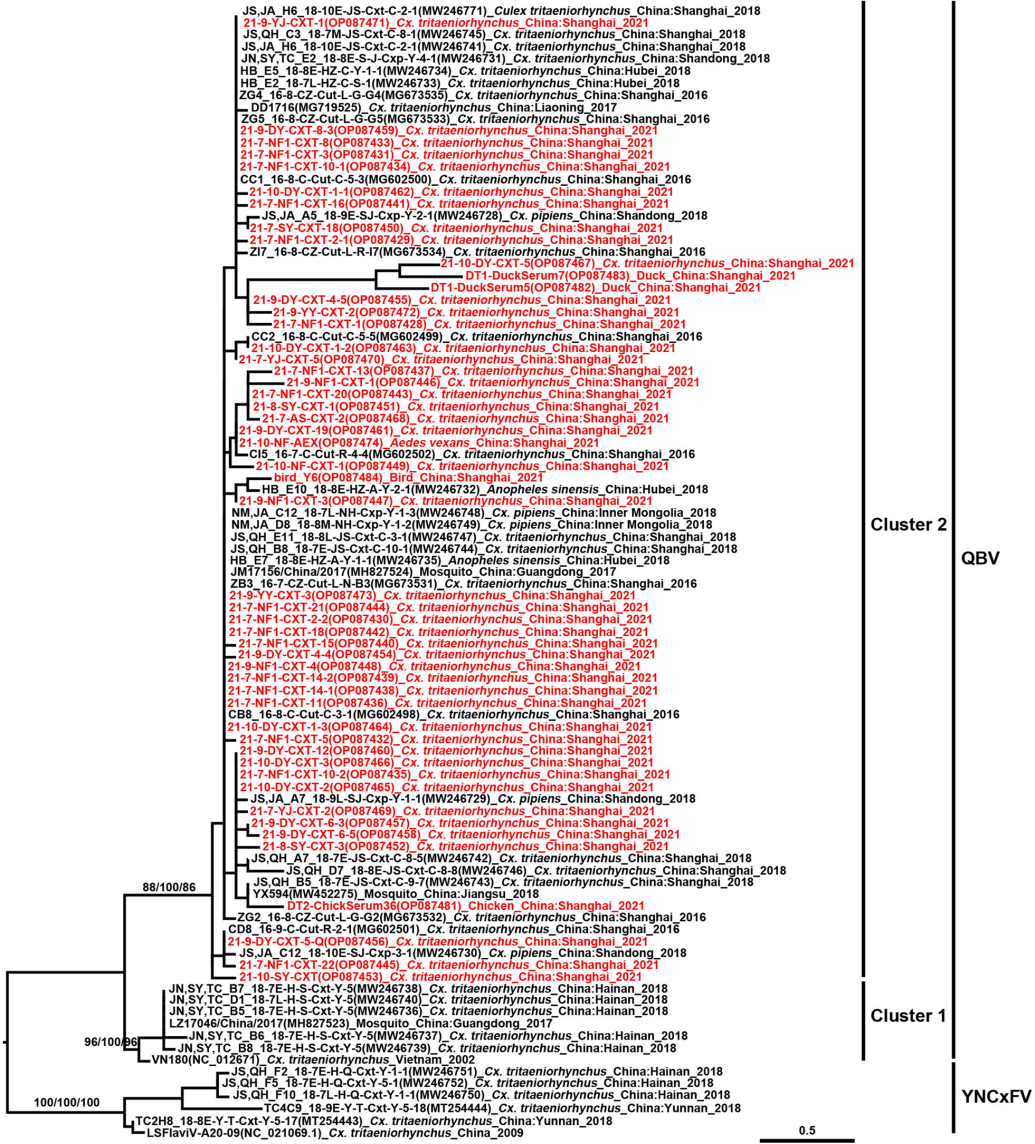
Maximum likelihood phylogenetic analysis of Quang Binh virus (QBV) partial non-structural 5 gene sequences. The strains of Yunnan Culex flaviviruses (YNCxFVs) are monophyletic, and paraphyletic to QBVs. The virus strain, GenBank accession number, host, collection country, and year are noted. The QBV sequences obtained in this study are marked in red. The numbers above each branch represent the bootstrap values (1000 replicates, not shown for less than 60%) of the maximum likelihood, Bayesian analyses, and neighbor-joining, respectively. The scale-bar indicates 0.5 substitutions per site

Newly detected QBVs from *Cx. tritaeniorhynchus* in Chongming Island were abundant and variable. It was also found in one pool of *Ae. vexans*. Their similarity in the conserved *NS5* gene ranged from 86.27 to 98.71%. Comparably, it was 90.87–97.71% among QBVs detected in poultry sera. In the *NS5* tree (Fig. 5), the QBVs can be clearly distinguished into two clusters. Cluster 1 was composed of the prototype VN180 obtained from *Cx. tritaeniorhynchus* collected in Vietnam in 2002, and strains mainly obtained from mosquitoes collected in southern China, such as Hainan and Guangdong provinces. Cluster 2 included strains from Shanghai, Hubei, Inner Mongolia, Shandong, Jiangsu, and Guangdong P/A/M. Remarkably, the Yunnan Culex flavivirus (YNCxFV) collected in Yunnan Province, which is located in southwestern China and neighboring Vietnam, was closely related, but paraphyletic with QBVs with high bootstrap value in the *NS5* tree (Fig. 5). All newly detected Chongming QBVs, both obtained from mosquito and avian samples, belonged to Cluster 2, based on phylogenetic analyses.

### Envelope protein sequence comparison on TMUV

Deduced amino acid sequences of E protein were aligned to compare Chongming mosquito (21-9-DY-CXT-5) and chicken (21-5-GY-8) strains, strains involved in 2010 TMUV outbreaks (FX2010), and the live attenuated vaccine strain FX2010-180P derived from FX2010 [38]. We observed a total of 23 mutation sites that were different between the Chongming TMUV strains and the FX2010 strain (Additional file 1:  Table S1). Notably, two of them were identical to the FX2010-180P strain - position 157, close to the glycosylation site 154, and position 312. None of mutations in N-linked glycosylation sites (103, 154, and 314) or histidines (144, 153, 163, 219, 246, 263, 285, 320, 398, and 443) [7, 9], were observed in Chongming TMUV strains. Notably, similar to most duck-origin TMUVs, the residue 156 for the E protein of Chongming strains is serine, which is critical for TMUV replication and transmissibility in ducks in the absence of mosquitoes [14].

### Arbovirus infection rates in mosquitoes

The infection rates (Table 2; Fig. 1) according to bias-corrected MLE and MIR for both TMUV and CHAOV in *Cx. tritaeniorhynchus* with 95% confidence intervals (*CI*), were 0.16 (95% *CI*: 0.03–0.51) and 0.16 (95% *CI*: 0.00–0.37) per 1000 individuals, respectively. However, the infection rates of QBVs varied at different sentinel sites. The bias-corrected MLE of the QBVs varies in different sentinel sites across the island, were with a mean value from 1.06 to 8.22 and an upper limit of 13.74 per 1000 *Cx. tritaeniorhynchus* (Table 2). The overall bias-corrected MLE infection rates of QBV in *Cx. tritaeniorhynchus* and *Ae. vexans*, were 3.92 (95%*CI*: 2.91–5.18) and 2.34 (95% *CI*: 0.14–11.22) per 1000 mosquitoes, respectively.

**Table 2:** Maximal likelihood estimation (MLE) and minimum infection rate (MIR) flaviviruses during mosquito activity season of Chongming Island, Shanghai in 2021

Detected virus	Survey areas		Host	No. individuals	No. PP	No. pools	Positive pool rate (%)	Bias corrected MLE (95% *CI*)	MIR (95% *CI*)
	Direction	Inhabitant							
TMUV	Eastern	Poultry farm	*Culex tritaeniorhynchus*	3154	2	65	3.08	0.64 (0.11–2.10)	0.63 (0.00–1.51)
	In total areas		*Cx. tritaeniorhynchus*	12,723	2	285	0.70	0.16 (0.03–0.51)	0.16 (0.00–0.37)
CHAOV	Eastern	Buffalo farm	*Cx. tritaeniorhynchus*	3194	1	71	1.41	0.31 (0.02–1.52)	0.31 (0.00–0.93)
		Cow farm	*Cx. tritaeniorhynchus*	3800	1	81	1.23	0.26 (0.02–1.28)	0.26 (0.00–0.78)
	In total areas		*Cx. tritaeniorhynchus*	12,723	2	285	0.70	0.16 (0.03–0.51)	0.16 (0.00–0.37)
QBV	Eastern		*Cx. tritaeniorhynchus*	10,148	40	217	18.43	4.33 (3.14–5.84)	3.94 (2.72–5.16)
		Buffalo farm	*Cx. tritaeniorhynchus*	3194	22	71	30.99	8.22 (5.31–12.30)	6.89 (4.02–9.76)
		Cow farm	*Cx. tritaeniorhynchus*	3800	4	81	4.94	1.06 (0.35–2.54)	1.05 (0.02–2.08)
		Poultry farm	*Cx. tritaeniorhynchus*	3154	14	65	21.54	4.95 (2.84–8.14)	4.44 (2.12–6.76)
	Central		*Cx. tritaeniorhynchus*	487	1	18	5.56	1.99 (0.12–9.64)	2.05 (0.00–6.07)
		Cow farm	*Cx. tritaeniorhynchus*	445	1	15	6.67	2.17 (0.13–10.51)	2.25 (0.00–6.65)
	Western		*Cx. tritaeniorhynchus*	2088	5	50	10.00	2.45 (0.93–5.40)	2.39 (0.30–4.49)
		Cow farm	*Cx. tritaeniorhynchus*	606	3	18	16.67	5.09 (1.40–13.74)	4.95 (0.00–10.54)
		Chicken farm	*Cx. tritaeniorhynchus*	1482	2	32	6.25	1.36 (0.25–4.45)	1.35 (0.00–3.22)
	In total areas		*Cx. tritaeniorhynchus*	12,723	46	285	16.14	3.92 (2.91–5.18)	3.62 (2.57–4.66)
	Eastern	Buffalo farm	*Aedes vexans*	383	1	14	7.14	2.49 (0.15–11.92)	2.61 (0.00–7.72)
	In total areas		*Ae. vexans*	409	1	22	4.55	2.34 (0.14–11.22)	2.44 (0.00–7.23)

*CI* Confidence intervals, *PP* Positive pools, *TMUV* Tembusu virus, *CHAOV* Chaoyang virus, *QBV* Quang Binh flavivirus

### Seroprevalence of TMUV-antibody in domestic poultry

To evaluate the prevalence of TMUV in Chongming, TMUV IgG antibodies were measured in domestic avian species (Fig. 1). TMUV-specific antibodies were widely present in chickens, ducks, and pigeons, with positivity rates of 50.31% (81/161), 52.22% (47/90), and 46.15% (18/39), respectively (Additional file 1:  Table S2). The seropositivity rates among the different avian species showed no significant differences (*P* = 0.437). However, there were marked differences (*P* = 0.026, < 0.05) among poultry farms of the three directions (Additional file 1:  Table S2). In eastern Chongming, near the Nature Reserve, it was 58.78% (77/131); in the central suburban area, it was 40.51% (32/79); and in the western extensive farming area, it was 46.25% (37/80).

## Discussion

### Spreading model and way of transmission of TMUV

The TMUV epidemic swept across coastal China unexpectedly with expanding speed, coverage, and economic loss for the duck industry. This was the first record of TMUV detected in China since its previous epidemic [6]. This unprepared epidemic of TMUV is probably due to the lack of the pathogen surveillance systems on detection in the vector and host. This is because before the epidemic, TMUV would experience transport, colonization, establishment, landscape spread in a novel habitat, according to the invasion theory [24, 41].

TMUV was previously identified in mosquitoes in Shandong, Yunnan, and Taiwan provinces in China [5, 8, 17]. In this study, we identified TMUV in *Cx. tritaeniorhynchus* in Shanghai. Wild birds are suspected to play a role in the spread of TMUV [20, 21]. Our findings provide robust evidence for the involvement of bird migration in the dispersion of TMUV since the virus has been detected in migratory birds, mosquitoes, and local poultry in Chongming. They have been shown to share high nucleotide sequence similarity, and generated a monophyletic clade with high bootstrap values in the phylogenetic trees (Figs. 2 and 3). The Chongming TMUV strains were distant from strains previously obtained from ducks in Shanghai but close to the TMUV strain that caused an outbreak in Guangdong in 2020 [10] and strains prevalent in Thailand, identified in mosquitoes and ducks [4, 42]. The coastal area of south-eastern China (Shanghai and Guangdong), and Thailand are on the route of East Asian–Australasian Flyway. The wetland ecosystem of Chongming Island serves as an important stopover site for migratory bird rests and energy supplements. Furthermore, 2–3 million migratory winter birds inhabit the wetland in spring and autumn [43], when vector mosquitoes are active in those region. Therefore, it provides an opportunity for pathogens to spread over long distances and colonize new habitats. The above findings demonstrate that the TMUV circulating in Chongming was probably imported from Southeast Asia by migratory birds, which then infected and transmitted between local mosquitoes and domestic avian species. The seroprevalence trend of TMUV in Chongming from the East to the West of the island supports this speculation further (Fig. 1). The seropositivity rate of TMUV-antibody was higher in avian sera collected from a private poultry farm near the Dongtan Nature Reserve than in those from other domestic poultry farms in Chongming Island. The positive rate of TMUV-antibody in the central region of Chongming was lower than that in the western part, probably due to the lower density of the farming industry in the central suburban area, while intensive animal husbandry in the western region facilitated the dispersion of TMUV.

Phylogenetic analyses of the *E* gene showed that TMUV is divided into three clusters. Cluster 1 mainly contains strains from Malaysia obtained from *Cx. tritaeniorhynchus* [2] and in chickens (strains: 1665/96, 3186/98, 4256/00, available on GenBank, unpublished data). The chicken-origin TMUV isolate, previously named Sitiawan virus, belongs to Cluster 1, which caused an outbreak in a broiler chicken farm in Malaysia with the symptoms of retarded growth, stretching of legs, or impairment of mobility [3]. Recently, TMUV (TMUV-TP1906) was isolated from *Cx. annulus* in Taiwan which is phylogenetically close to the Sitiwan strain and belongs to Cluster 1 [17]. Notably, TMUV-TP1906 grew well in C6/36 and Vero cells without noticeable cytopathic effects (CPE) but caused obvious CPE in DF-1 chicken fibroblast cell lines [17].

From the three subclusters of Cluster 2 of TMUV (Fig. 3), except for Cluster 2.1, other clusters have been recorded in both mosquitoes and avian species. Strains belonging to Cluster 2.2. dominated the 2010 TMUV epidemic by non-vector transmission [13, 15] and sporadically induced outbreaks in China thereafter [7, 8]. To date, Cluster 2.2 has been specific to China. Most strains belonging to Cluster 2.3, which emerged later than Cluster 2.2. were of duck-origin. However, it drew attention for its high mortality rate (50%) in goslings and even showed severe neurological dysfunction in affected ducklings and goslings compared to strains of Cluster 2.2 [44]. This high virulence cluster has covered southeast coast (Zhejiang and Guangxi Provinces), central (Hubei Provinces), southwest (Chongqing Municipality), and northeast China. Although it has a short history, Cluster 2.3 was involved in the 2013 TMUV outbreak in Cherry Valley ducks in Chongqing [45], the 2019 TMUV outbreak in Jingding ducks in northeast China [46], and the same year, it caused an outbreak in goslings in Anhui Province [44]. Based on the TMUV *E* gene tree (Fig. 3), TMUV Cluster 2.2 has been isolated in China since 2010 and has expanded rapidly in more than half of China over a few years. However, it is interesting to note that Cluster 2.3 seems to arise as the dominant sub-cluster of most TMUV strains isolated in China after 2015. This phenomenon is in agreement with observations that Cluster 2.3 was the primary subcluster of TMUV circulating in Thailand duck farms during 2015–2017 [42]. Whether Cluster 2.2 will be replaced by the high virulence Cluster 2.3, as the genotype replacement of Japanese encephalitis virus Genotype III to Genotype I [47], requires close surveillance and further study.

Cluster 3 is considered a novel cluster of TMUVs [42], although it (ThCar) was discovered in Thailand in the late of last century [3]. Information on the phenotypic and virulence features of Cluster 3 is limited. Recently, it (CTLN strain) caused a decrease in egg production in laying hens in southwest Guangdong Province and showed significant antigenic differences with the JXSP strain, which belongs to Cluster 2.2 [10]. Furthermore, Cluster 3 strain (CTLN strain) replicates much more effectively in mosquito cell line, as compared with the performance of Cluster 2.2 (JXSP strain); however, no differences were observed in BHK-21 and avian cell lines (CEF and DEF) [10]. The potential threat of Cluster 3 to avian hosts is probably underestimated. In the Guangdong 2020 TMUV outbreak, the seropositivity rate of TMUV antibody in affected hen flocks was 95.83–100%, as determined by ELISA [10, 48]. The daily egg production rate declined from ca. 80% to ca. 50% in 32 to 40 weeks old infected hens and most of them recovered at three weeks post-infection with no mortality [10]. Moreover, the virus spread to the flocks in the neighboring farms and the affected flocks showed similar clinical manifestations, though it did not appear to significantly decrease the daily egg production rate [10]. Consistently, the highest seropositive rates of TMUV antibody were observed in chicken and duck sera (85.19 and 93.33%, respectively), collected from a poultry farm near Dongtan National Nature Reserve (Fig. 1) with no case reported. The poultry industry in Chongming Dongtan is famous for its organic ground chickens and ducks reared in an ecological organic environment, free of industrial contamination; thus, the reared poultry can used for both egg laying or selling as broiler chickens. High seropositive rates in avian hosts and moderate reduction in egg production caused by Cluster 3 can be considered as potential reasons which explain that in spite of a relatively high seropositive rate of TMUV-antibody in avian specimens, clinical cases of TMUV have not been recorded in Chongming until now. In view of the virologic and pathological features of Cluster 3, it is likely that the strains have acquired attenuated pathogenicity compared to those of Cluster 2, as the Omicron variant of SARS-CoV-2 [49].

### The expansion of ISFVs, and its co-infection with mosquito-borne viruses

The present study revealed that the composition and infection rate of mosquito-borne viruses were dynamic and more severe in Chongming than in our previous surveillance performed five years ago [25]. The dual-host ISFV, CHAOV was found in *Cx. tritaeniorhynchus* collected in Shanghai, which is far from its original geographic region [50]. In addition to the expansion of the distribution of QBV, the infection rate of QBV in mosquitoes has increased in Chongming in recent five years. It was 8.22 (95% *CI*: 5.31–12.30) per 1000 *Cx. tritaeniorhynchus* collected in 2021 at the sentinel site close to the Dongtan National Reserve, four times more than that obtained in the sample place in 2016, which was 1.97 (95% *CI*: 0.74–4.37). The mechanism of QBV expansion in China and its side effects warrant further investigation [51]. Here, QBV was found in the same mosquito pool, infected with TMUV. Notably, it was detected in the serum samples of chickens and ducks in Chongming, and even in throat swab samples of migratory birds. This is contradictory to our common knowledge on insect-specific flaviviruses, which can only replicate in mosquito cells and not in mammalian cells [52]. Further studies on the physiological and pathogenic characteristics of QBV are necessary to clarify this extraordinary phenomenon.

The main limitation of the study is that the presence of QBV in avian samples betrays our recognition on ISFV, which needs further supports though virus isolation from avian samples or the replication of QBV in vertebrate cells. The diversity and infection rate of mosquito-borne virus in Chongming mosquitoes probably be underestimated by RT-PCR. Study on meta-transcriptomic analysis could be conducted to reveal the spectrum of local mosquito virome in further. Seroepidemiological survey of TMUV by neutralization test can be performed to provide a more robust result on the characterization of TMUV infection among domestic poultry than that of ELISA.

## Conclusions

Our findings demonstrated the circulation of mosquito-borne zoonotic viruses in Chongming. The presence of TMUVs in migratory birds, mosquitoes, and domestic poultry, with high sequence homology, indicated a possible model of long-distance spreading of TMUV from Southeast Asia to Chongming Island. Thus, mosquitoes may be involved in the transmission of TMUV in Chongming. More likely, non-vector transmission among avian species plays a more dominant role in virus colonization in domestic poultry due to the low infection rate of TMUV in mosquitoes, but high seroprevalence of TMUV-antibody in local avian species. These findings expand our understanding of the mechanism of TMUV spread and transmission in vector and avian species, providing additional knowledge for the development of strategies for the prevention and control of TMUV. The dual-host ISFV, CHAOV, was first observed in Shanghai, far from its original geographic region. Moreover, the expansion and increasing tendency of natural infection with QBV have been recorded in recent years. The potential threat of ISFVs and prospects to use it against mosquito-borne pathogen thereby need close attention and further study. The present study revealed that the composition and infection rate of mosquito-borne viruses were dynamic and more severe in Chongming than in our previous surveillance performed five years ago. This highlights the necessity of sustainable and systematic mosquito-borne pathogen surveillance for the early warning, risk evaluation, and prevention of emerging mosquito-borne viruses.

## Supplementary Information

**Additional file 1**: **Table S1**. Polymorphic residues in the E protein among Chongming Tembusu virus strains, FX2010 strain, and the live attenuated vaccine strain FX2010-180P derived from FX2010. Table S2. Seroprevalence of Tembusu virus antibodies in different poultries and directions in Chongming Island in 2021.

## Data Availability

All data generated or analysed during this study are included in the published article and Additional file.

## Abbreviations

CHAOV: Chaoyang virus
MLE: Maximum likelihood estimation
MIR: Minimum infection rate
NJ: Neighbour-joining
ISFV: Insect-specific flavivirus
QBV: Quang Binh flavivirus
TMUV: Tembusu virus
YNCxFV: Yunnan Culex flavivirus.
