# Supplementary material for: Long-distance spread of Tembusu virus, and its dispersal in local mosquitoes and domestic poultry in Chongming Island, China
Source: Infect Dis Poverty. 2023 May 22;12:52. doi: 10.1186/s40249-023-01098-9 (PMC10200701; doi:10.1186/s40249-023-01098-9)
Supplement: Supplementary file 1 — Additional file 1: Table S1. Polymorphic residues in the E protein among Chongming Tembusu virus strains, FX2010 strain, and the live attenuated vaccine strain FX2010-180P derived from FX2010. Table S2. Seroprevalence of Tembusu virus antibodies in different poultries and directions in Chongming Island in 2021. [file 40249_2023_1098_MOESM1_ESM.docx]

Additional file 1: Table S1. Polymorphic residues in the E protein among Chongming Tembusu virus strains, FX2010 strain, and the live attenuated vaccine strain FX2010-180P derived from FX2010

| Position on E protein | 21-9-DY-CXT-5 | 21-5-GY-8 | FX2010 | FX2010-180P |
| --- | --- | --- | --- | --- |
| 38 | R | R | K | K |
| 69 | S | S | T | T |
| 72 | P | P | S | S |
| 89 | D | D | E | G |
| 91 | V | V | I | I |
| 135 | I | I | V | V |
| 150 | N | N | S | S |
| 157 | V | V | A | V |
| 180 | L | L | M | M |
| 185 | A | A | T | T |
| 236 | R | R | K | K |
| 304 | I | M | M | M |
| 312 | A | A | V | A |
| 328 | S | T | S | S |
| 332 | S | S | T | T |
| 346 | W | L | L | L |
| 349 | K | M | M | K |
| 358 | I | I | V | V |
| 365 | P | P | S | S |
| 371 | V | V | I | I |
| 391 | E | E | G | G |
| 394 | K | K | R | R |
| 467 | V | V | A | A |

Additional file 1: Table S2. Seroprevalence of Tembusu virus antibodies in different poultries and directions in Chongming Island in 2021

|  | Sample size | Positive no. (%) | Negative no. (%) | Odds ratio (95% CI) |
| --- | --- | --- | --- | --- |
| Species |  |  |  |  |
| Chickens | 161 | 81 (50.31) | 80 (49.69) | 0.937 (0.790–1.110) |
| Ducks | 70 | 39 (55.71) | 31 (44.29) | 1.164 (0.784–1.727) |
| Pigeon | 59 | 26 (44.07) | 33 (55.93) | Reference |
| Total | 290 | 146 (50.34) | 144 (49.66) | P=0.420 |
| Directions |  |  |  |  |
| Eastern | 131 | 77 (58.78) | 54 (41.22) | 1.213 (0.975–1.510) |
| Western | 80 | 37 (46.25) | 43 (53.75) | 0.732 (0.518–1.035) |
| Central | 79 | 32 (40.51) | 47 (59.49) | Reference |
| Total | 290 | 146 (50.34) | 144 (49.66) | P=0.026 <0.05 |
